# Oligomerization of Optineurin and Its Oxidative Stress- or E50K Mutation-Driven Covalent Cross-Linking: Possible Relationship with Glaucoma Pathology

**DOI:** 10.1371/journal.pone.0101206

**Published:** 2014-07-01

**Authors:** Jie Gao, Masafumi Ohtsubo, Yoshihiro Hotta, Shinsei Minoshima

**Affiliations:** 1 Department of Photomedical Genomics, Basic Medical Photonics Laboratory, Medical Photonics Research Center, Hamamatsu University School of Medicine, Hamamatsu, Japan; 2 Department of Ophthalmology, Hamamatsu University School of Medicine, Hamamatsu, Japan; University of Iowa, United States of America

## Abstract

The optineurin gene, *OPTN*, is one of the causative genes of primary open-angle glaucoma. Although oligomerization of optineurin in cultured cells was previously observed by gel filtration analysis and blue native gel electrophoresis (BNE), little is known about the characteristics of optineurin oligomers. Here, we aimed to analyze the oligomeric state of optineurin and factors affecting oligomerization, such as environmental stimuli or mutations in *OPTN*. Using BNE or immunoprecipitation followed by sodium dodecyl sulfate-polyacrylamide gel electrophoresis (SDS-PAGE), we demonstrated that both endogenous and transfected optineurin exist as oligomers, rather than monomers, in NIH3T3 cells. We also applied an *in situ* proximity ligation assay to visualize the self-interaction of optineurin in fixed HeLaS3 cells and found that the optineurin oligomers were localized diffusely in the cytoplasm. Optineurin oligomers were usually detected as a single band of a size equal to that of the optineurin monomer upon SDS-PAGE, while an additional protein band of a larger size was observed when cells were treated with H_2_O_2_. We showed that larger protein complex is optineurin oligomers by immunoprecipitation and termed it covalent optineurin oligomers. In cells expressing *OPTN* bearing the most common glaucoma-associated mutation, E50K, covalent oligomers were formed even without H_2_O_2_ stimulation. Antioxidants inhibited the formation of E50K-induced covalent oligomers to various degrees. A series of truncated constructs of *OPTN* was used to reveal that covalent oligomers may be optineurin trimers and that the ubiquitin-binding domain is essential for formation of these trimers. Our results indicated that optineurin trimers may be the basic unit of these oligomers. The oligomeric state can be affected by many factors that induce covalent bonds, such as H_2_O_2_ or E50K, as demonstrated here; this provides novel insights into the pathogenicity of E50K. Furthermore, regulation of the oligomeric state should be studied in the future.

## Introduction

The optineurin gene, *OPTN*, has been identified as a causative gene of adult-onset primary open-angle glaucoma (POAG) [1] and was named after optic neuropathy-inducing protein. Glaucoma is the second leading cause of blindness worldwide [2]–[4]. It is a neurodegenerative disease of the optic nerve, characterized by the progressive loss of retinal ganglion cells (RGCs) and their axons. The most common form of glaucoma is POAG, affecting over 33 million people throughout the world [2]. One subgroup of POAG is normal tension glaucoma (NTG). The molecular mechanism of glaucoma is largely unknown; however, numerous studies have established a genetic etiology for this disorder.

It is a common view that glaucoma is a multifactorial disease resulting from the involvement of genetic variation in one or more genes and/or various environment factors. The environment factors include higher intraocular pressure (IOP) and other stimuli mainly associated with IOP or age, such as oxidative stress [5] and cytokines [6]. Sequence alterations in *OPTN* were found in 16.7% of families with autosomal dominantly inherited NTG. The most prevalent mutation was the missense p.Glu50Lys (E50K) mutation, which occurred in 13.5% of these families [1]. In addition, E50K-bearing glaucoma patients appear to have more progressive and severe phenotypes [7], [8].

Optineurin (OPTN) is a 577-amino-acid protein, which contains several putative functional domains, including at least one leucine zipper, multiple coiled-coil domains, a ubiquitin-binding domain (UBD), and a zinc finger domain; the latter two domains are located at the C terminal region [9]. Public databases show that OPTN homologs from mouse, rat, pig, and bovine have a substantial degree of similarity to human OPTN. Moreover, OPTN shares 53% sequence similarity with the NF-κB essential modulator (NEMO), and was therefore also named NEMO-related protein [10]. OPTN expression has been demonstrated in several non-ocular tissues, such as the brain, heart, kidney, and skeletal muscle [11], as well as in ocular tissues, including the trabecular meshwork, retina, and non-pigmented ciliary epithelium [12]. Endogenous OPTN was previously reported to locate largely to the Golgi complex [10], [13], but a later report revealed that it is also present in the cytoplasm, with a diffuse distribution pattern [14].

To date, several functions of OPTN have been explored, such as negative regulation of TNFα-induced NF-κB activation [15], [16], Golgi organization [17], [18], vesicle trafficking [19], [20], regulation of metabotropic glutamate receptor signaling [21], antiviral signaling [22], autophagic clearance of protein aggregates [23], and regulation of gene expression [24]. Those functions are always fulfilled by the interaction with optineurin-binding proteins, including polyubiqutinated receptor-interacting protein (polyUb RIP) [15], deubiquitinase CYLD [25], Rab8 [17], [26], [27], huntingtin [26], myosin VI [17], transferrin receptor (TfR) [19], metabotropic glutamate receptor [21], TANK-binding kinase 1 (TBK1) [22], and transcription factor IIIA [28].

The mechanism by which the most typical glaucoma-associated mutation in OPTN, E50K, contributes to glaucoma has always been unclear. It has been shown that the E50K selectively induces the death of RGCs and that this effect is not shared by three other OPTN mutations, H26D, H486R, and R545Q [29]. This phenomenon by E50K was mediated through oxidative stress [29] and apoptosis [30]. E50K transgenic mice showed marked apoptosis and degeneration of the entire retina, which was due to disruption of the interaction between optineurin and Rab8 [31]. Moreover, E50K mutation impairs trafficking of TfR due to altered interactions with Rab8 and TfR [19], [20]. Recently, it was found that E50K mutant strongly interacted with TBK1, which evoked intracellular insolubility of OPTN, leading to improper OPTN transition from the endoplasmic reticulum to the Golgi body [32]. A clearer understanding of the detailed characteristics of the E50K mutation in glaucoma pathogenesis requires further study.

Despite the numerous efforts to investigate OPTN function, there are limited studies providing structural information, in particular about the oligomeric state of OPTN, which is crucial for the function of OPTN. To date, several studies have suggested that OPTN is present in high molecular weight complexes in cultured cells [10] or is able to oligomerize [14], [32]. In this study, we confirmed the self-oligomerization of OPTN in cultured cells, and to our knowledge, this study is the first to provide data regarding visualization of the oligomerization of OPTN in cells. Moreover, we found that OPTN trimerizes upon H_2_O_2_ stimulation and identified that the UBD region of OPTN is essential for its trimerization. We also determined the relationship between the oligomeric state of OPTN and the E50K mutation, which provides new insights into E50K-mediated pathogenesis.

## Materials and Methods

### cDNA cloning

The coding region of human *OPTN* cDNA was amplified by polymerase chain reaction (PCR), using retina marathon cDNA (TAKARA, Japan) library as the template. The open reading frame size of *OPTN* is 1734 bp, based on the nucleotide sequence information obtained from RefSeq, NCBI (NM 001008211). PCR primers containing the recognition sites for restriction endonuclease *Bsa*Ι and *Eco*RΙ were used: sense 5′-TTCCACggtctcAAGCTTATGTCCCATCAACCTCTCAG-3′ and antisense 5′-ATACATgaattcTTAAATGATGCAATCCATCA-3′. The resulting PCR products were digested with *Bsa*Ι and *Eco*RΙ and inserted into a p3×FLAG-CMV-7.1 expression vector (Sigma-Aldrich Japan, K.K., Tokyo, Japan), which was pre-cut with *Hin*dIII and *Eco*RΙ. The sequence of the p3×FLAG/OPTN (FLAG-OPTN) construct was verified by DNA sequencing.

For generating GFP-OPTN, pAcGFP1-C3 vector (Clontech Laboratories, Inc., Mountain View, CA, USA) was digested with *Hind*III and FLAG-OPTN was partially digested with *Hin*dIII. The digested vectors underwent a ligation reaction, then PCR was performed to amplify the ligated products using the following pair of primers: sense 5′-GAATTCTGCAGTCGACGGTA-3′ (GFP-Fwd) and antisense 5′-TTAAATGATGCAATCCATCA-3′ (OPTN-Rev). The PCR products were ligated to form closed circle plasmids containing the complete *OPTN* coding sequence. Sequencing was performed to confirm the nucleotide sequences of pAcGFP1-C3/OPTN (GFP-OPTN).

### Expression plasmids and DNA transfection

All mutants were created using the KOD-Plus Mutagenesis Kit (TOYOBO, Japan). The names of the mutant constructs and the corresponding primers used for their amplification were described in Table 1.

**Table 1:** List of the primers for generating OPTN mutants.

Glaucoma-associated mutants:		
	forward	Reverse
H26D	5′-ACCTGGCCCACCCAAACCT-3′	5′-CGGGGGGTCCATTTCCTGTG-3′
E50K	5′-AAGAACCACCAGCTGAAAGAA-3′	5′-GGTCAGGAGCTCTTTCATCTG-3′
M98K	5′-GGCCTTGAGTCATGAGAATGAG-3′	5′-TTTAGACGCTCTTTTGCTTCTTT-3′
H486R	5′-GTGAGGAAAAGGAGCAACTGGC-3′	5′-GAATTTTCTCTCTCGCTGCTC-3′
R545Q	5′-GCAACAGCGGAATATTCCGA-3′	5′-TGCCAGTCCCTGTCCTCAGC-3′
**A designed mutant:**		
D474N	5′-AATTTTCATGCTGAAAGAGCAGCGAG-3′	5′-AGAACAGTAAACTTCCATCTGAGCCC-3′
**The truncated mutants:**		
Lc1st (Lc1-120)	5′-TCATCTGAGGACCCCACTGATG-3′	5′-AAGCTTGTCATCGTCATCCTTGTAATC-3′
Lc2nd (Lc121-287)	5′-GAAGAGAAAGGCCCGGAGA-3′	5′-CCTTTCTGATTTCCCTTTTAG-3′
Lc3rd (Lc288-422)	5′-CTGAAGGAACTGAGTGAAAAACTGGA-3′	5′-ATCATTCTCTTTCTCTGTGCTCCC-3′
Lc4th (Lc423-577)	5′-TAAGAATTCATCGATAGATCTGATATCGG-3′	5′-CACTGCCCTGTCCACTTTTTCTG-3′
LcUBD (Lc424-509)	5′-GGCAGGCAGTCCTTGATGGAGATGC-3′	5′-CAGCACTGCCCTGTCCACTTTTTCTGAC-3′
LcZF (Lc552-577)	5′-TAAGAATTCATCGATAGATCTGATATCGG-3′	5′-CGGAATATTCCGCTGTTGCCGCCAG-3′

The primers were from Operon Biotechnologies. All mutants were verified by DNA sequencing.

Constructs were transfected into NIH3T3 or HeLaS3 cells using FuGENE6 (Promega, Madison, WI, USA) according to the manufacturer's instructions. Usually, 24 h after transfection, cells were washed with 1× PBS (−) and then collected for the subsequent procedures.

### Cell cultures and drug treatments

NIH3T3 and HeLaS3 cells were cultured at 37°C in a 5% CO_2_ atmosphere in Dulbecco's modified Eagle's medium (DMEM; Sigma) supplemented with 10% fetal bovine serum, 100 units/ml penicillin, and 100 mg/ml streptomycin.

Cells were stimulated with 25 mM H_2_O_2_ (WAKO, Japan) for 20 min or 10 ng/ml TNFα (WAKO) for 5 h. The antioxidants applied in this study included N-acetyl-l-cysteine (NAC; WAKO) and l(+)-ascorbic acid (WAKO). NAC at various concentrations (2.5, 25, 250, 2500 *µ*M) or ascorbic acid (0.1, 1, 10, 100 *µ*M) was added to the culture medium at 4 h after E50K transfection. Afterwards, every 12 h, the medium was replaced with new medium containing antioxidants. E50K-expressing cells, without any treatment, but with medium replacement, were used as a control.

### Antibodies

The antibodies used in this study included rabbit anti-OPTN antibody (Cat. No. 10837-1-AP; ProteinTech Group, Inc., Chicago, IL, USA), mouse anti-FLAG M2 monoclonal antibody (F3165; Sigma-Aldrich), rabbit anti-GFP antibody (MBL International Corporation, Woburn, MA, USA), monoclonal anti-α-tubulin antibody (T8203; Sigma-Aldrich), goat anti-mouse IgG/HRP (P0447; DAKO, Tokyo, Japan), goat anti-rabbit IgG/HRP (P0448; DAKO), and rabbit anti-goat IgG/HRP (P0449; DAKO), and AlexaFluor488-conjugated donkey anti-mouse IgG (A21202; Invitrogen, Carlsbad, CA, USA).

### *In situ* proximity ligation assay (PLA)

HeLaS3 cells were cultured on coverslips and were co-transfected with p3×FLAG/OPTN and pAcGFP1-C3/OPTN plasmids, while cells co-transfected with p3×FLAG/MYOC and pAcGFP1-C3/OPTN were used as negative controls. After 24 h of transfection, the cells were rinsed in 1× PBS(–) and fixed in 4% paraformaldehyde (PFA) for 20 min, followed by permeabilization in 0.5% TritonX-100 for 5 min and blocking in 1% Perfect Block for 30 min. After incubation with mouse anti-FLAG and rabbit anti-GFP primary antibodies for 1 h at room temperature, cells were treated with secondary antibodies, anti-mouse Ig conjugated with a specific oligonucleotide (PLA probe PLUS) and anti-rabbit Ig with another specific oligonucleotide (PLA probe MINUS). AlexaFluor488-conjugated donkey anti-mouse IgG was used to detect FLAG-tagged OPTN. Then, according to the *in situ* PLA instruction manual (Olink Bioscience, Uppsala, Sweden), the ligation solution was added and the oligonucleotides hybridized to the two PLA probes to form a closed circle when in close proximity. After subsequent addition of the amplification solution, the rolling-circle amplification (RCA) reaction took place to generate a product with a repeated sequence, which was hybridized by the fluorescently labeled oligonucleotides. PLA hybridization signals were observed using a fluorescent microscope (Axioskop 2 plus; Zeiss, Gottingen, Germany).

### Blue native gel electrophoresis

The blue native gel electrophoresis (BNE) was performed according to the instructions for the NativePAGE Novex Bis-Tris Gel System (Invitrogen). NIH3T3 cells transfected with or without p3×FLAG/OPTN were lysed using 1× NativePAGE Sample Buffer (50 mM BisTris, 6 N HCl, 50 mM NaCl, 10% w/v glycerol, 0.001% Ponceau S, pH 7.2) with addition of 1% n-dodecyl-β-d-maltoside (DDM). NativePAGE G-250 sample additive, at a final concentration of 0.2%, was added to the protein samples immediately prior to electrophoresis. Then, the prepared samples were loaded onto a NativePAGE 4–16% BisTris Gels. Electrophoresis was performed with ice-cold 1× NativePAGE Anode Buffer (50 mM BisTris, 50 mM Tricine, pH 6.8) and ice-cold Dark Blue Cathode Buffer (0.02% G-250, 50 mM BisTris, 50 mM Tricine), at 150 V for 20 min. Next, the Dark Blue Cathode Buffer was replaced by the Light Blue Cathode Buffer (0.002% G-250, 50 mM BisTris, 50 mM Tricine) and electrophoresis continued for an additional 90 min.

The proteins in the gel were transferred to Immobilon-P membrane (MILLIPORE, Chelmsford/Billerica, MA) in the 1× NuPAGE Transfer Buffer (25 mM Bicine, 25 mM Bis-Tris, 1 mM EDTA, 0.05 mM chlorobutanol) with 10% methanol/gel at 30 V for 1 h. Endogenous OPTN was detected with rabbit anti-OPTN polyclonal antibody (Catalog No. 10837-1-AP, ProteinTech Group, Inc.) and horseradish peroxidase (HRP)-conjugated goat anti-rabbit secondary antibody (P0448; DAKO). FLAG-tagged OPTN was detected by mouse anti-FLAG M2 monoclonal antibody (F3165; Sigma-Aldrich Japan, K.K.) and goat anti-mouse IgG/HRP (P0447; DAKO).

### Immunoprecipitation

Cells were lysed with RIPA buffer (50 mM Tris-HCl, pH 7.5, 150 mM NaCl, 1 mM EDTA, 1% Triton X-100) supplemented with protease-inhibitor cocktail (Roche, Mannheim, Germany). Next, the appropriate antibody and Protein G Sepharose 4 Fast Flow (GE Healthcare, Piscataway, NJ, USA) were added to the cell lysate; this was then washed in RIPA buffer 3 times immediately prior to use. The mixture was rotated uniformly at 4°C continuously for overnight to allow antigen-antibody binding. After washing the immunoprecipitates with RIPA buffer, the proteins were eluted from the beads with 1× sample buffer (0.24 M Tris-HCl, 40% v/v glycerol, 8% w/v SDS, 20% v/v 2-mercaptoethanol (ME), 0.01% w/v BPB) and were then boiled at 95°C for 5 min. The supernatant was loaded onto a 10–20% polyacrylamide gel for western blotting, as described below.

### Western blotting

Crude cell lysate or immunoprecipitated proteins were subjected to SDS-PAGE under reducing conditions. The proteins in the gel were transferred to an Immobilon-P membrane (MILLIPORE) in a semi-dry apparatus at 1.2 mA/cm^2^ for 1 h. Then, the membrane was soaked in 5% skim milk to block non-specific binding of antibodies, followed by incubation with the primary antibody diluted in PBST (1× PBS +0.1% v/v Tween 20). After washing the membrane with PBST for several times, HRP-conjugated secondary antibody was applied at a 1∶5000 dilution. Immunoreactive protein bands were detected by luminescence using ImmunoStar reagents (Wako), and the luminescent images were analyzed using an LAS1000mini system (Fuji Film, Tokyo, Japan).

### Dual-luciferase reporter assay

HeLaS3 cells were cultured in 24-well plates for 24 h and co-transfected with three plasmids, including two luciferase reporter vectors, pGL4.32[*luc2P*/NF-κB-RE/Hygro] (0.09 µg) and pGL4.74[*hRluc*/TK] (0.01 µg), along with 0.1 µg of one of the other plasmids, per well. Cells transfected with empty FLAG vector and the two reporter vectors were used as controls. After 20 h of transfection, the cells were treated with 10 ng/ml TNFα for an additional 5 h. Subsequently, the cells were lysed and the activities of firefly and *Renilla* luciferases were measured sequentially for each sample, using the Dual-Luciferase Reporter Assay System (DLRAS; Promega). Each DLRAS experiment was repeated at least 3 times. Data with n ≥3 for each experiment were statistically analyzed using Dunnett's test and shown as the mean ± standard deviation. *P*<0.05 was considered statistically significant.

## Results

### OPTN mainly exists in high molecular weight protein complexes under physiological condition

**Figure 1:**
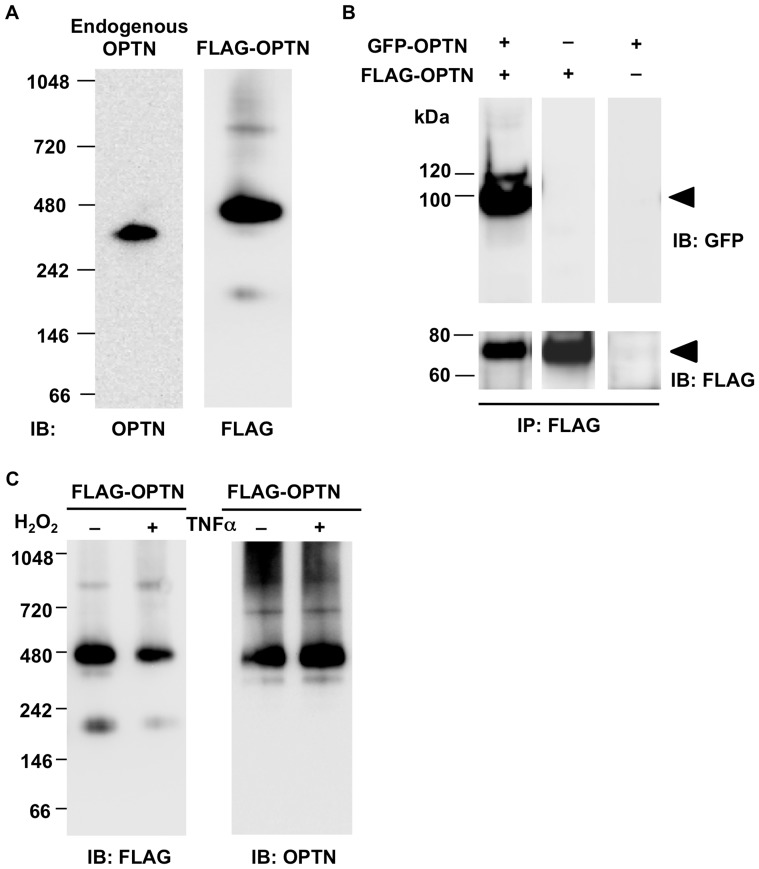
In cultured cells, optineurin exists mainly as oligomers, which are not dissociated by apoptotic stimuli. **A.** Lysates from NIH3T3 cells transfected with or without FLAG-OPTN were analyzed by blue native gel electrophoresis and western blotting (WB). The anti-OPTN or anti-FLAG antibody was used to detect endogenous optineurin (OPTN; left lane, 374 kDa) or the transfected OPTN (right lane, 470 kDa), respectively. In both cases, OPTN is mainly present within a high molecular protein complex (HMC). **B**. NIH3T3 cells were co-transfected with GFP-OPTN and FLAG-OPTN, while cells transfected with GFP-OPTN or FLAG-OPTN were used as controls. Cell lysates were immunoprecipitated with the anti-FLAG antibody and then subjected to WB with the anti-GFP antibody (upper panel) or the anti-FLAG antibody (lower panel). GFP-tagged OPTN (upper panel, left lane) was detected in immunoprecipitates from co-transfected cell lysates, but not in either control. **C**. NIH3T3 cells expressing FLAG-OPTN were treated with 25 mM H_2_O_2_ for 20 min (+), or 10 ng/ml TNFα (+), or left untreated (−). After blue native gel electrophoresis followed by WB, FLAG-tagged OPTN was detected mainly at approximately 470 kDa with either anti-FLAG or anti-OPTN antibody, in all cases. There was no obvious difference in protein bands between treated and untreated groups. IB: immunoblotting; IP: immunoprecipitation.

As previously reported [10], [14], both endogenous and transfected OPTN forms oligomers or high molecular weight protein complexes (HMCs) as detected by BNE or gel filtration analysis. The amount of oligomers or OPTN-containing HMC was markedly increased compared to that of OPTN monomers, which led us to consider in what state OPTN exists under physiological conditions. To investigate the native form of OPTN under physiological conditions, lysates of NIH3T3 cells were analyzed by BNE under non-reducing conditions. A single band was detected at approximately 374 kDa by an antibody directed against OPTN (Fig. 1A, left lane), indicating that OPTN is at least present in, or may be the only component of the HMC.

A similar result was observed when human OPTN tagged with FLAG was expressed, via plasmid transfection, in NIH3T3 cells. Although multiple bands were seen, with size ranging from 220 to 870 kDa, FLAG-OPTN was mainly present in the HMC with a size of 470 kDa (Fig. 1A, right lane).

**Figure 2:**
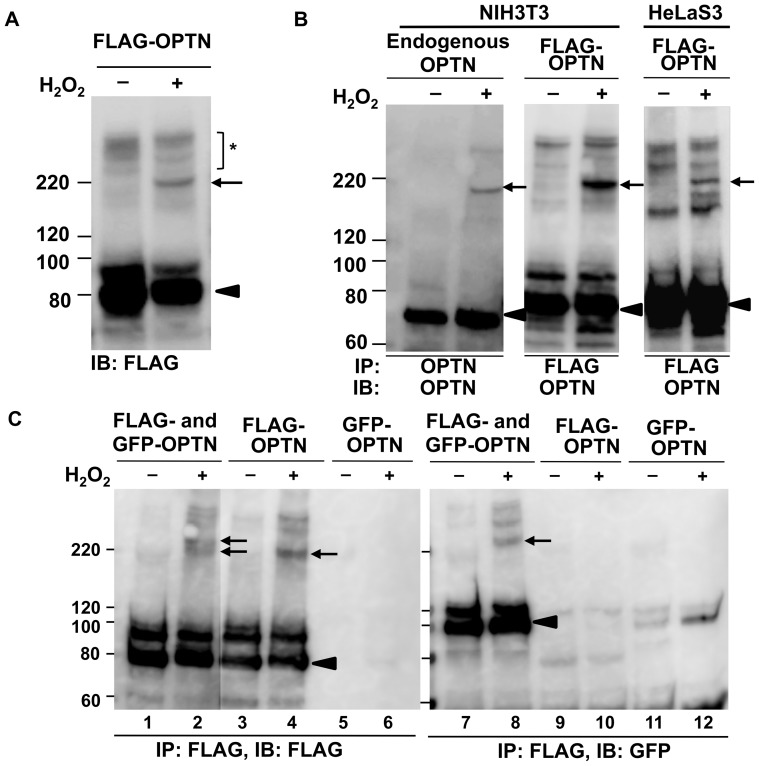
Covalently bonded oligomers of optineurin are induced by oxidative stress. **A.** NIH3T3 cells transfected with FLAG-OPTN were incubated without or with 25 mM H_2_O_2_ for 20 min and then analyzed by SDS-PAGE and western blotting with anti-FLAG antibody. A protein complex (right lane, arrow) was observed in the H_2_O_2_-treated sample. Other high molecular weight bands (asterisk) were seen at low density in both untreated and H_2_O_2_-treated samples. The optineurin (OPTN) monomer was observed at the expected position (arrowhead) **B**. After standard transfection into NIH3T3 or HeLaS3 cells and treatment without or with FLAG-OPTN and H_2_O_2_, lysates were immunoprecipitated with anti-OPTN antibody for detection of endogenous OPTN (left panel), or anti-FLAG antibody for detection of transfected OPTN. The H_2_O_2_-induced protein band was detected by anti-OPTN antibody in all cases (arrow). Both endogenous and transfected OPTN monomer band was observed at an expected position (arrowhead). Another band was seen above the monomer in the lane of transfected OPTN, but it was not analyzed in this study because it was not present in the case of endogenous OPTN. **C**. NIH3T3 cells expressing GFP-OPTN and FLAG-OPTN, or both, were treated without or with H_2_O_2_. Cell lysates were immunoprecipitated with anti-FLAG antibody and analyzed by western blotting with antibodies against FLAG (lanes 1–6) or GFP (lanes 7–12). The H_2_O_2_-induced bands were detected in co-transfected and FLAG-transfected samples (lanes 2, 4, and 8, arrows). OPTN monomer bands were shown as arrowheads. IB: immunoblotting; IP: immunoprecipitation.

Considering the native conformation of the protein, the size estimation by BNE may have an approximately 15% error. In this case, the estimated mass of the HMC of endogenous OPTN may range between 320 and 430 kDa, and the HMC of FLAG-OPTN between 400 and 540 kDa. The molecular weights of the endogenous and FLAG-tagged OPTN monomer were 67.9 kDa and 74.8 kDa, respectively, as estimated from the SDS-PAGE results shown in Fig. 2B. Comparison of the molecular sizes of the HMCs and the OPTN monomer indicated that the HMC may be an OPTN hexamer. Since this conclusion was drawn merely based on the molecular size estimation from BNE results, which as mentioned above has non-negligible error, we cannot exclude the possibility that the HMC contains molecule(s) other than OPTN. However, our results suggested that, under physiological conditions, the majority of OPTN is present in the HMC and that the HMC may be or may contain homo-oligomers of OPTN.

### Oligomers of transfected OPTN are diffusely located in the cytoplasm

To establish whether OPTN interacts with itself to form oligomers, NIH3T3 cells were co-transfected with pAcGFP1-C3/OPTN and p3×FLAG/OPTN. Cell lysates were immunoprecipitated with mouse monoclonal anti-FLAG antibody, and then subjected to western blotting and detected by rabbit polyclonal anti-GFP antibody (Fig. 1B, left lane). When cells transfected with GFP-OPTN or FLAG-OPTN were used as controls, the GFP-OPTN band was not detected (Fig. 1B, middle and right lanes, the position of the arrowhead). These results indicate that OPTN interacts with itself to form oligomers. Together with the previous results [10], [14], [32], this implied that the majority of OPTN exists as oligomers that are contained in the HMC.

**Figure 3:**
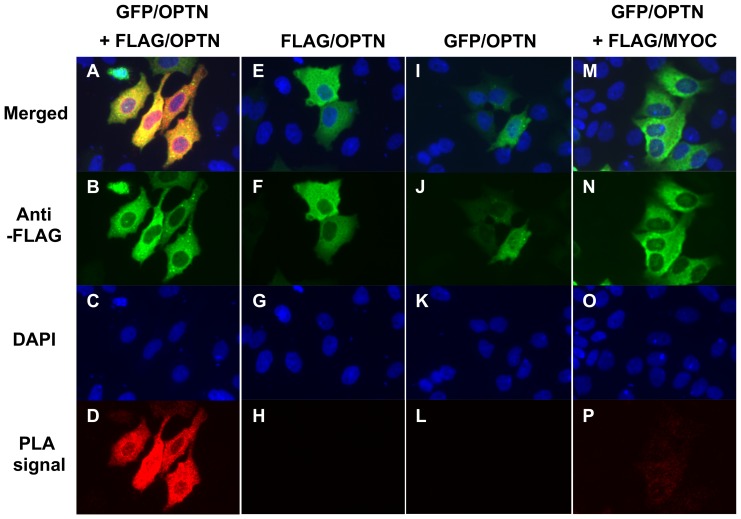
Self-interaction of optineurin as visualized using an *in situ* proximity ligation assay in HeLaS3 cells. HeLaS3 cells were co-transfected with GFP- and FLAG-tagged optineurin (OPTN) (**A**–**D**), or transfected with FLAG-tagged OPTN (**E**–**H**), GFP-tagged OPTN (**I**–**L**) or co-transfected with GFP-tagged OPTN along with FLAG-tagged myocilin (**M**–**P**). The latter three groups were used as negative controls. **A**, **E**, **I** and **M**: merged images of **B**–**D**, **F**–**H**, **J**–**K**, and **N**–**P**, respectively. **B**, **F**, **J**, and **N**: staining with anti-FLAG antibody. **C**, **G**, **K** and **O**: DAPI staining of nuclei. **D**, **H**, **L**, and **P**: signals detected by *in situ* proximity ligation assay (PLA). Strong PLA signals were observed only in HeLaS3 cells expressing GFP- and FLAG-tagged OPTN (**D**), indicating interaction between GFP- and FLAG-tagged OPTN.

We hypothesized that if OPTN exists as oligomers in cells under physiological conditions, the subcellular localization of the oligomers should be consistent with previous reports which showed that OPTN was localized diffusely in the cytoplasm and was partially associated with the Golgi apparatus [14], [18]. Thus, we next investigated the subcellular localization of OPTN oligomers. We applied the *in situ* PLA, which not only detects the localization of proteins but also enables visualization of protein interaction in fixed cells. HeLaS3 cells cultured on cover slips were co-transfected with AcGFP1-C3/OPTN and p3×FLAG/OPTN. Single transfection of AcGFP1-C3/OPTN or p3×FLAG/OPTN and co-transfection of p3×FLAG/MYOC and AcGFP1-C3/OPTN were performed as negative controls because it was found that myocilin did not interact with OPTN. Strong PLA signals were observed throughout the cytoplasm when cells were co-transfected with AcGFP1-C3/OPTN and p3×FLAG/OPTN (Fig. 3D), which revealed that GFP-tagged OPTN interacted with FLAG-tagged OPTN to form oligomers that had a diffuse, cytoplasmic distribution. No significant PLA signals were seen in controls (Fig. 3H, L, and P). Based on the PLA protocol, the negative reaction should not necessarily be completely blank; hence, the weak signals detected with co-transfection of p3×FLAG/MYOC and AcGFP1-C3/OPTN did not represent interaction between these proteins. These results together suggested that self-oligomerized OPTN exists ubiquitously in the cytoplasm.

### OPTN oligomers are not dissociated by apoptotic stimuli

We next considered whether the oligomeric state of OPTN might be affected by extracellular stimuli, leading to dissociation of OPTN oligomers. To answer this question, we examined the effect of H_2_O_2_ and TNFα on OPTN oligomerization, since such stimuli have been proven to be increased in glaucoma patients and in turn contribute to retinal ganglion cells apoptosis [33]–[38]. NIH3T3 cells transfected with p3×FLAG/OPTN were stimulated with 25 mM H_2_O_2_ for 20 min or with 10 ng/ml TNFα for 5 h; the cell lysates were then subjected to BNE followed by western blotting under non-reducing conditions. Untreated cells expressing FLAG-tagged OPTN were used as a control. No distinct differences were observed between treated and untreated samples (Fig. 1C). Similar results were obtained with endogenous OPTN (data not shown). These results together suggested that OPTN oligomers are not dissociated by these apoptotic stimuli.

However, it is known that overexpression of OPTN in NIH3T3 cells protects cells from H_2_O_2_-induced cell death [39]. The inhibitory effect of OPTN on TNFα-induced NF-κB activation is also well known [15], [16]. Our observation that OPTN oligomers were not dissociated in the stimulated cells indicated that OPTN exerts its functions in its oligomeric form when cells experience apoptotic stimuli; thus, the oligomer may be the functional form of OPTN.

### Covalently bonded OPTN oligomers are induced by oxidative stress

Using SDS-PAGE, the overwhelming majority of endogenous OPTN and FLAG-tagged OPTN in NIH3T3 cells were observed at sizes of 67.9 kDa and 74.8 kDa, respectively (Fig. 2B, arrowheads), equivalent to the size of the OPTN monomer. Physiologically existing OPTN oligomers were thus dissociated into monomers in cultured cells under reducing conditions, which indicated that OPTN molecules present in an oligomer are joined by noncovalent or disulfide bonds.

Surprisingly, we noted that an additional OPTN-containing band appeared above the OPTN monomer band in the lysates of OPTN-transfected NIH3T3 cells that had been stimulated with H_2_O_2_ (Fig. 2A, right lane, arrow). This band, with a size of 214 kDa, was found after SDS-PAGE with a reducing reagent (2-mercapto ethanol), suggesting that the protein in the 214-kDa complex are linked together by covalent bonds other than disulfide bonds (non-SS covalent bond). Other high molecular weight bands were also seen, although at lower intensity (Fig. 2A, asterisk), but those bands were not clearly different between H_2_O_2_-treated and untreated cells, suggesting that those complexes were not induced by H_2_O_2_ treatment. Therefore, we only focused on the 214-kDa band, which represents a dynamic change in the OPTN-containing protein complex in response to oxidative stress.

To confirm the above result, we analyzed endogenous OPTN as well as transfected OPTN in NIH3T3 cells and HeLaS3 cells, using immunoprecipitation to facilitate observation of the H_2_O_2_-induced band. Cells transfected with or without FLAG-OPTN were incubated with 25 mM H_2_O_2_ for 20 min at 37°C. Cell lysates were immunoprecipitated with anti-OPTN antibody for endogenous OPTN and anti-FLAG antibody for transfected OPTN, and the immunoprecipitates were analyzed by SDS-PAGE and western blotting with anti-OPTN antibodies. The expected H_2_O_2_-induced band was observed in both untransfected and transfected cells in the presence of H_2_O_2_ (Fig. 2B, arrows). The estimated molecular mass of H_2_O_2_-induced band in case of endogenous OPTN was 204.9 kDa which was in close agreement with three times of endogenous OPTN monomer (67.9×3 = 203.7; ratio  = 3.02) (Fig. 2B, left panel, arrow and arrowhead; Table 2, the line of “Endogenous”). In case of transfected FLAG-tagged OPTN, the H_2_O_2_-induced band was 214 kDa in size which was also close to three times of FLAG-tagged OPTN monomer (74.8×3 = 224.4; ratio  = 2.86) (Fig. 2B, middle panel; Table 2, the line of “WT”). This result suggested that if this protein complex contained only OPTN, it may be an OPTN trimer cross-linked by non-SS covalent bonds.

**Table 2:** Predicted and estimated molecular weights of monomers and covalent oligomers of optineurin and its mutants.

		Estimated Mw based on WB results (kDa)		
OPTN	Predicted Mw (kDa)	Monomer (a)	Covalent optineurin oligomer (b)	Ratio (b:a)
**Endogenous**	**67.0**	**67.9**	**204.9**	**3.02**
**WT**	**68.8**	**74.8**	**213.7**	**2.86**
**Lc1st**	**54.9**	**59.8**	**178.5**	**2.98**
**Lc2nd**	**50.2**	**51.6**	**157.5**	**3.05**
**Lc3rd**	**53.2**	**56.8**	**167.7**	**2.95**
**Lc4th**	**50.9**	**57.5**		

OPTN, optineurin; WT, wild-type; Lc1st, lacking the first OPTN region; L2nd, lacking the second OPTN region; Lc3rd, lacking the third OPTN region; Lc4th, lacking the fourth OPTN region.

We next tested whether this covalent protein complex contained two or more OPTN molecules. We firstly co-transfected GFP-OPTN and FLAG-OPTN into NIH3T3 cells, alongside negative controls of the single transfection of GFP-OPTN or FLAG-OPTN. After treatment with (or without) H_2_O_2_, cells were lysed and immunoprecipitated with anti-FLAG antibody and western blotted using anti-FLAG or anti-GFP antibody. As shown in lane 8 of Fig. 2C (the band indicated by the arrow), the covalent protein complex immunoprecipitated by the anti-FLAG antibody was immunoreactive to anti-GFP antibody in the H_2_O_2_-treated group, suggesting that at least one molecule of FLAG-tagged OPTN and one molecule of GFP-tagged OPTN were present in the protein complex.

The protein complex detected by the anti-GFP antibody (lane 8, Fig. 2C, arrow) appeared to be larger than that detected by the anti-FLAG antibody in lysates of cells transfected with FLAG-OPTN alone (lane 4, Fig. 2C, arrow). The larger size of the protein complex detected by the anti-GFP antibody in cells co-transfected with GFP-OPTN and FLAG-OPTN (lane 8, Fig. 2C) is probably due to the larger size of the GFP-tag, compared to the FLAG-tag. The co-transfection of both kinds of tagged OPTN should produce two types of protein complexes, one which contains GFP-OPTN and another which does not contain it, and both of them should be detected by anti-FLAG antibody. Indeed, this protein complex appeared as double bands (lane 2, Fig. 2C, arrows).

**Figure 4:**
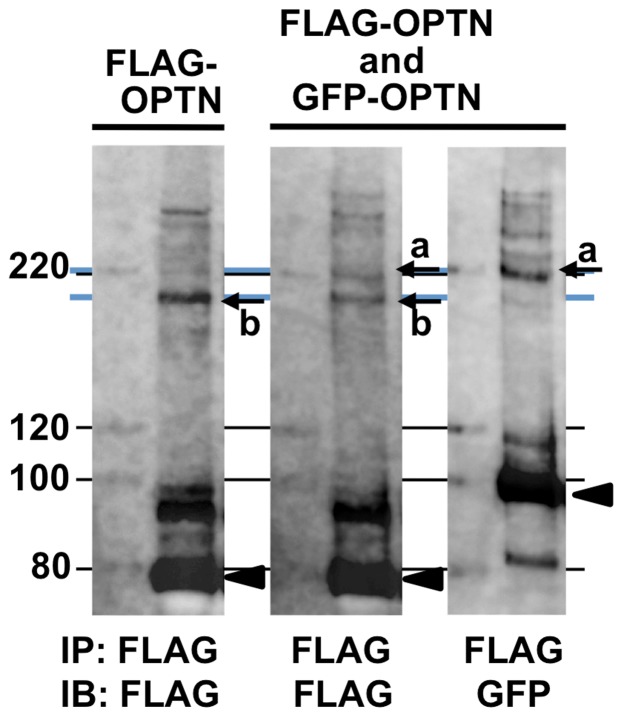
Higher resolution analysis of covalently bonded optineurin oligomers produced by co-transfection of FLAG-OPTN and GFP-OPTN. NIH3T3 cells expressing FLAG-OPTN, or both FLAG-OPTN and GFP-OPTN, were treated with H_2_O_2_. Cell lysates were immunoprecipitated with anti-FLAG antibody and subjected to SDS-PAGE, on a 7.5% acrylamide gel, followed by western blotting with anti-FLAG or anti-GFP antibodies. In co-transfected cell lysates, the anti-FLAG antibody detected two bands (middle panel, arrows a and b), while the anti-GFP antibody detected only the higher molecular weight band (right panel, arrow a). In lysates of cells transfected with FLAG-OPTN alone, only the lower molecular weight band was detected (left panel, arrow b). OPTN monomer bands are shown as arrowheads. The blue lines were drawn as a guide to each of the corresponding H_2_O_2_-induced bands. NIH3T3 cells expressing FLAG-OPTN were treated without or with H_2_O_2_. IB: immunoblotting; IP: immunoprecipitation.

To more accurately analyze these double bands, we repeated the above experiment as those of lanes 2, 4, and 8 in Fig. 2C and electrophoresed the immunoprecipitation products alongside molecular weight markers on a polyacrylamide gel of lower concentration (7.5%), which is capable of higher resolution of bands in this high molecular weight region. This yielded clear results wherein two bands were obtained upon anti-FLAG western blotting of lysates of co-transfected cells (bands “a” and “b” in the middle panel of Fig. 4); the anti-GFP western blot of co-transfected cells showed only the higher molecular weight band (band “a” in the right panel of Fig. 4), while the anti-FLAG western blot of lysates of cells transfected with FLAG-OPTN alone showed only the lower molecular weight band (band “b” in the left panel of Fig. 4). These results strongly suggested that the lower of the two bands in cells co-transfected with FLAG-OPTN and GFP-OPTN consisted of only FLAG-OPTN and that the higher molecular weight band also contained GFP-OPTN. These results strongly suggested that the protein complex contained two or more OPTN molecules and that these are cross-linked by non-SS covalent bonds.

### E50K mutation induces the autonomous formation of non-SS covalent bonded optineurin oligomers, which are inhibited by anti-oxidants

**Figure 5:**
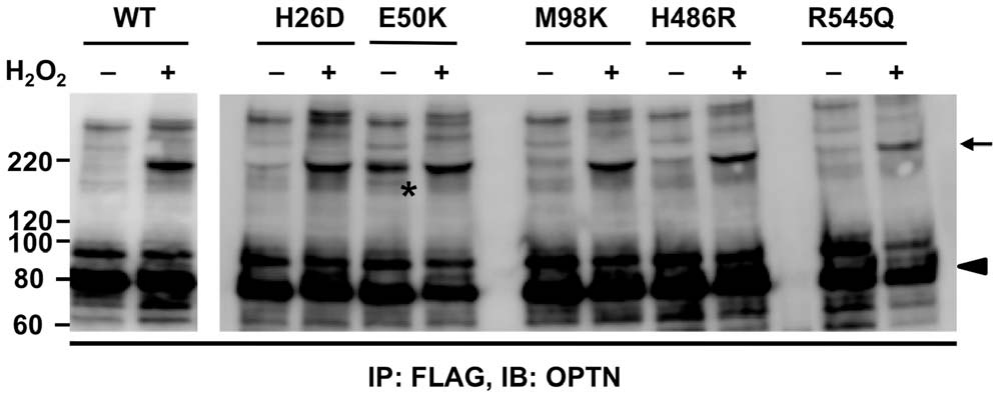
Expression of the E50K mutant of optineurin induces formation of covalent optineurin oligomers. NIH3T3 cells were transfected with FLAG-OPTN (WT), or optineurin (OPTN) harboring the H26D, E50K, M98K, H486R, or R545Q mutations, separately. After 70 h of transfection, cells were treated without or with 25 mM H_2_O_2_ for 20 min. Cell lysates were immunoprecipitated with anti-FLAG antibody and immunoprecipitates were analyzed by SDS-PAGE and western blotting with anti-OPTN antibody. Covalent OPTN oligomers (arrow) were observed in all the H_2_O_2_-treated cells, as well as in the untreated cells expressing the E50K mutant (asterisk). OPTN monomers were shown as arrowhead. IB: immunoblotting; IP: immunoprecipitation.

We next examined the influence of glaucoma-associated OPTN mutations on OPTN oligomerization, as well as the type of bonds formed. NIH3T3 cells transfected with plasmids expressing wild-type (WT) OPTN or its mutants (H26D, E50K, M98K, H486R, and R545Q) were treated without or with H_2_O_2_. Cell lysates were immunoprecipitated with anti-FLAG antibody and subjected to SDS-PAGE followed by western blotting with anti-OPTN antibody. Interestingly, although 214-kDa bands were observed in all the H_2_O_2_-treated groups (Fig. 5, arrow), it was also clearly detected in the case of the E50K mutant in the absence of H_2_O_2_ treatment (Fig. 5, asterisk). E50K itself induced the formation of covalent OPTN oligomers, which suggested that E50K may produce similar intracellular conditions as H_2_O_2_ treatment of cells, which we have discussed later in this report.

**Figure 6:**
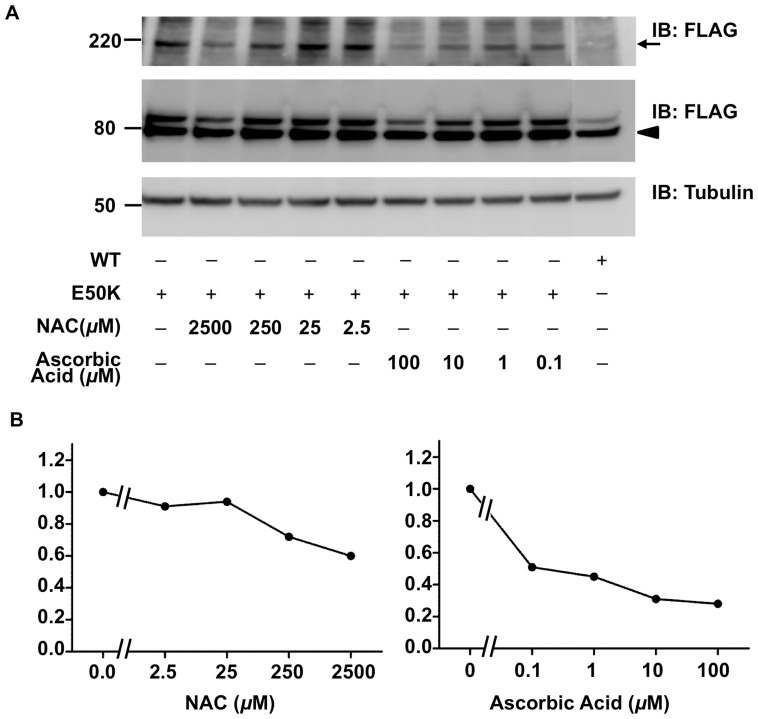
Antioxidants have an inhibitory effect on E50K-induced covalent oligomers of optineurin. **A.** N-acetyl-cysteine (NAC), or ascorbic acid was added to the culture medium of NIH3T3 cells after 4 h of E50K-construct transfection. Detailed drug treatment is described in Materials and Methods. Cells were lysed after 64 h of transfection and lysates were analyzed by SDS-PAGE and western blotting with anti-FLAG (upper and middle panels) and anti-tubulin antibodies (lower panel). Covalent optineurin oligomers and monomers were shown as arrow and arrowhead, respectively. **B**. The degree of inhibitory effect was expressed as a ratio. The density of every band shown in A was measured by the software Multi Gauge (Version 3.0, Fuji Photo Film Co., Ltd). The ratio is determined as [oligomer/(monomer + oligomer) of the anti-oxidant treated sample]/[oligomer/(monomer + oligomer) of the untreated E50K-expressing sample]. Therefore, the ratio of the untreated E50K expressing sample is 1.

To determine whether E50K induces the formation of covalent OPTN oligomers by increasing intracellular oxidative stress, we examined the ability of antioxidants to inhibit E50K-induced covalent OPTN oligomers. We applied NAC or ascorbic acid at various concentrations to treat NIH3T3 cells expressing E50K OPTN. This resulted in different degrees of reduction in the formation of covalent OPTN oligomers, in a dose-dependent manner (Fig. 6A and B). In particular, the incubation with 0.1 mM ascorbic acid consistently resulted in greater than 70% reduction in the amount of E50K-induced covalent OPTN oligomers. These results supported the hypothesis that E50K increases intracellular oxidative stress [29], which probably induces the formation of covalent OPTN oligomers.

### The non-SS covalent-bonded OPTN oligomers are trimers

**Figure 7:**
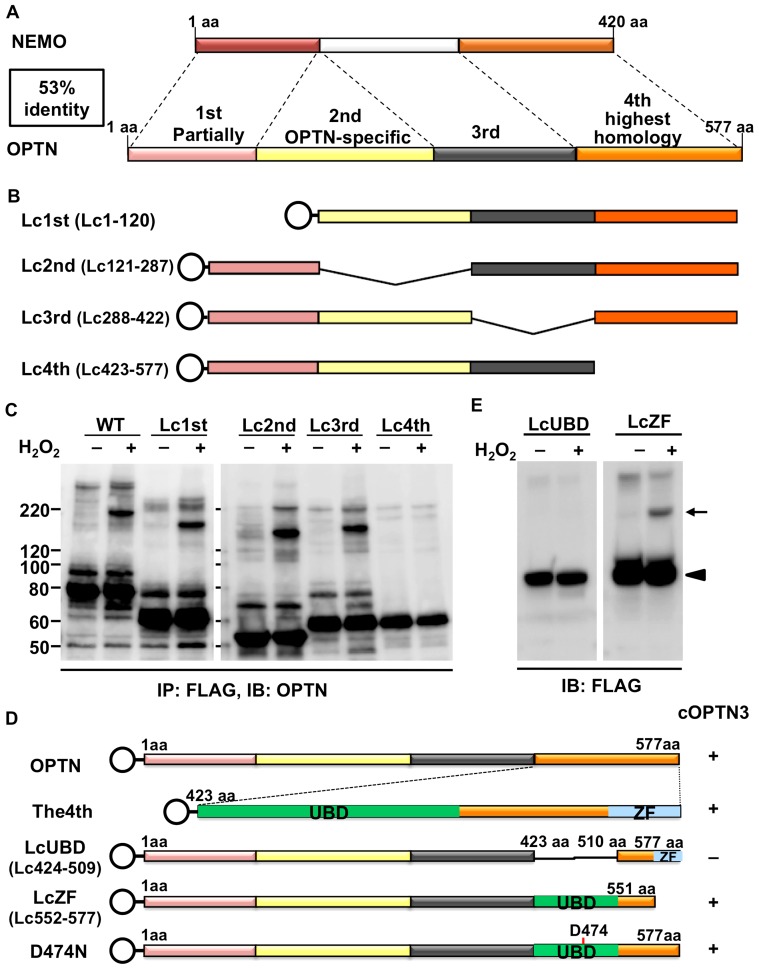
Covalent optineurin trimers are formed via the ubiquitin binding domain upon oxidative stress. **A.** Schematic representation of the sequence comparison result between optineurin (OPTN) and NF-κB essential modulator (NEMO). The OPTN amino acid sequence was divided into four regions based on its degree of homology with NEMO. **B**. Plasmids for four truncation mutants (each lacking one of the regions), tagged with N-terminal FLAG (shown as open circle), were constructed, and named Lc1st, Lc2nd, Lc3rd, and Lc4th. **C**. Lysates from NIH3T3 cells expressing wild-type (WT) or one of the truncated constructs and incubated without or with H_2_O_2_ (25 mM, 20 min) were immunoprecipitated with anti-FLAG antibody and then analyzed by SDS-PAGE and western blotting (WB) with anti-OPTN antibody. H_2_O_2_-induced covalent oligomers were detected in cells expressing WT, Lc1st, Lc2nd, and Lc3rd, but not Lc4th. The estimated molecular weights from the WB results are described in Table 2, indicating that covalent oligomers may be OPTN trimers. The WT lanes in the left panel are the same as the left panel of Fig. 2B. **D**. Two deletion constructs lacking the ubiquitin-binding domain (UBD) or the zinc finger domain in the 4th region, as well as a point-mutated construct, were made and named LcUBD, LcZF, and D474N. **E**. SDS-PAGE and WB analysis showed that the covalent trimers were formed in LcZF-transfected cells, but not in the cells expressing LcUBD.

As the above results indicated that one of the possible deleterious effects of E50K might involve the formation of covalent OPTN oligomers, it was necessary to study its features. We determined the protein functional domain of OPTN responsible for the formation of the covalent OPTN oligomers. OPTN shares 53% sequence homology with NEMO and was previously named NEMO-related protein [10]. The sequence homology between these two proteins might explain their similar behaviors, such as competitively binding with RIP [15] and nuclear translocation upon different stimuli [40], [41]. Moreover, the ability of NEMO to form oligomers has also been studied previously [42]–[44]. Therefore, we decided to use four plasmid clones each of which has one of four deletions in the OPTN coding sequence, which we previously reported [45]. The four deletion regions had been decided according to its homology with NEMO (Fig. 7A). The 4th region (residues 423–577) with the highest NEMO homology, the 2nd region (residues 121–287), which is not present in NEMO, and the 1st and 3rd regions (residues 1–120 and 288–422, respectively) with low homology to NEMO, were selected for deletion and the corresponding deletion clones were named Lc1st (lacking the 1st region), Lc2nd, Lc3rd, and Lc4th (Fig. 7B). The predicted protein sizes of the FLAG-tagged truncated OPTN expressed from these four plasmids are 54.9, 50.2, 53.2, and 50.9 kDa, respectively.

NIH3T3 cells expressing WT or any of the four truncated OPTNs were incubated without or with H_2_O_2_. The cell lysates were immunoprecipitated with anti-FLAG antibody followed by western blotting with anti-OPTN antibody. Cross-linking of the OPTN-containing complex with non-SS covalent bonds were induced by H_2_O_2_ treatment in WT OPTN, as well as in Lc1st, Lc2nd, and Lc3rd, but not in Lc4th (Fig. 7C). We estimated the molecular weight of each of the OPTN-containing covalent complexes and the OPTN monomer based on these immunoblot results using molecular weight markers (Table 2). To our surprise, the data, together with the molecular weights estimated from above results of endogenous OPTN, showed that the molecular size of each OPTN-containing covalent complex was close to triple the size of its corresponding monomer (Table 2, “Ratio” column). We found that there were at least two OPTN molecules present in the OPTN-containing covalent complex (Fig. 2C and 4), and the result in Fig. 7C was obtained simply by designed truncation of the OPTN molecule. If there were other molecule(s) besides OPTN contained in the covalent oligomers, the total molecular weight of the other component(s) would have to be constant in each complex. However, the subtraction results of [size of a covalent oligomer] – 2× [size of the corresponding monomer] for Endogenous, WT, Lc1st, Lc2nd and Lc3rd are 69.1, 64.1, 58.9, 54.3, and 54.1, respectively, which are far from a constant value and probably abolish the possibility of the involvement of the other molecule(s) in the covalent oligomers. These results together strongly suggest that the OPTN-containing covalent complexes are OPTN trimers (subsequently termed cOPTN_3_) that are cross-linked by non-SS covalent bonds; moreover, the 4th domain of OPTN is necessary for cOPTN_3_ formation.

Additionally, we detected another FLAG-tag-positive band, which was 15–20 kDa larger than the monomer of intact or truncated OPTN, in WT, Lc1st, Lc2nd, and Lc3rd samples, but not in Lc4th samples (Fig. 7C). It is possible that this extra FLAG-tag-positive band was produced by some type of post-translational modification. Given the size of this band, SUMOylation is one of the possibilities which typically results in an approximate 17-kDa increase in molecular weight, although other types of modification cannot be ruled out. It appears as if the 4th region (or even the narrower UBD region) is required for the production of the extra band (Fig. 7C and 7E). However, since the extra band did not appear in the case of endogenous OPTN (Fig. 2B), it may be an artifact.

### The UBD of OPTN is responsible for cOPTN_3_ formation in a ubiquitin-independent manner

We next narrowed down the 4th region of OPTN to further clarify the domain involved in cOPTN_3_ formation. There are two important domains in the 4th region, the UBD (residues 424–509) and the zinc finger motif (ZF, residues 552–577). As shown in Fig. 7D, we generated a FLAG-tagged UBD truncated construct (LcUBD) and a ZF truncated construct (LcZF). After treating NIH3T3 cells expressing LcUBD or LcZF with or without H_2_O_2_, cell lysates were immunoblotted with anti-FLAG antibody. cOPTN_3_ complexes were not observed when OPTN lacked the UBD, even with stimulation by H_2_O_2_, while cOPTN_3_ complexes were still formed when the ZF was lacking (Fig. 7E), which suggested that the UBD of OPTN is responsible for the formation of cOPTN_3_.

**Figure 8:**
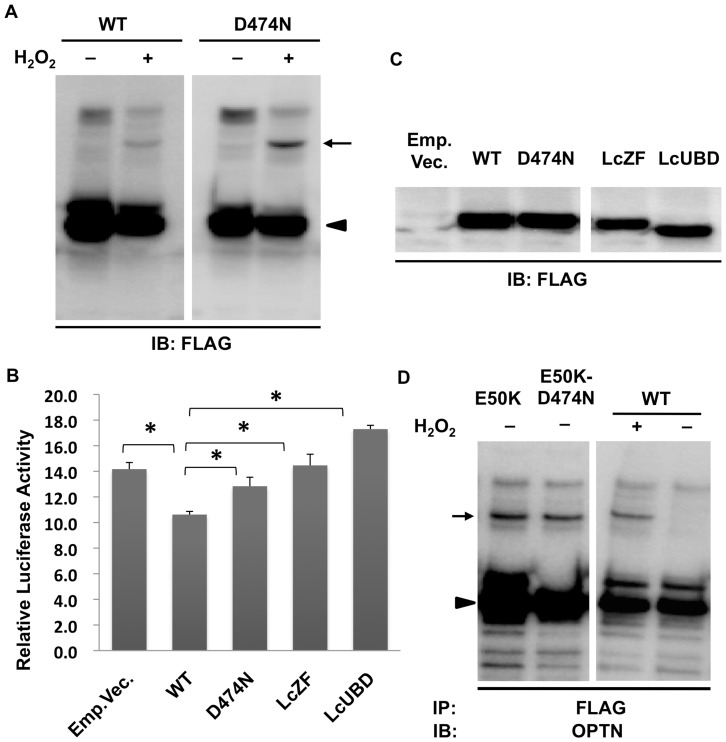
Ubiquitin binding is not involved in the formation of covalent optineurin trimers. **A.** Covalent trimers were observed in NIH3T3 cells expressing the D474N mutant (arrow) using SDS-PAGE followed by western blotting (WB). Monomers were shown as arrowhead. **B**. HeLa cells transfected with wild-type (WT) or one of the mutants of optineurin (OPTN; D474N, LcZF, or LcUBD) along with firefly and *Renilla* luciferase reporters were incubated with 10 ng/ml TNFα for 5 h and examined using a Dual-Luciferase Reporter Assay System (DLRAS). Cells expressing WT OPTN or empty vector were used as controls. The ratios of firefly luminescence to *Renilla* luminescence represented the relative luciferase activity (n = 3). There were statistically significant differences between WT and mutants of OPTN (*P*<0.05). **C**. Confirmation of the expression of WT and OPTN mutants in HeLaS3 cells before DLRAS, shown by WB. **D**. Expression of double point mutations of OPTN (E50K–D474N) induced the formation of covalent trimers in NIH3T3 cells, without H_2_O_2_ treatment. IB: immunoblotting; IP: immunoprecipitation.

It was previously shown that the D474N mutation in the UBD of OPTN abolished its binding to ubiquitin, thus abolishing the inhibitory effect of OPTN on TNFα-induced NF-κB activation [15]. We observed that the D474N mutant was still able to form cOPTN_3_ in the presence of H_2_O_2_ (Fig. 8A). A dual-luciferase reporter assay was used to examine the inhibitory effect of the D474N mutant, as well as that of LcUBD and LcZF on TNFα-induced NF-κB activation. HeLa cells transfected with WT or either of these mutants along with the firefly and *Renilla* luciferase reporters were treated with TNFα. As expected, LcUBD and the D474N mutant did not inhibit TNFα-induced NF-κB activation. Interestingly, LcZF also abolished the inhibition of TNFα-induced NF-κB activation (Fig. 8B). The expression of WT and mutant OPTN was confirmed by western blotting (Fig. 8C). Since it has been clearly demonstrated that the D474N mutation abolishes the inhibition of TNFα-induced NF-κB activation by abrogating the ubiquitin-binding ability of OPTN [15], [16], the ability of the D474N mutant to form cOPTN_3_ complexes suggested that cOPTN_3_ are formed in a ubiquitin-independent manner.

To further confirm this observation, we constructed a double mutant OPTN, E50K–D474N. We previously showed that the E50K mutant induced cOPTN_3_, probably by increasing intracellular oxidative stress; since the D474N mutation did not abolish H_2_O_2_-induced cOPTN_3_ formation, it would also not abolish E50K-induced cOPTN_3_ formation. As expected, cOPTN_3_ complexes were still formed without H_2_O_2_ stimulation in cells expressing the E50K–D474N mutant (Fig. 8D). These observations together suggested that the UBD is responsible for the formation of cOPTN_3_, but ubiquitin binding itself is unnecessary, suggesting another function of the UBD.

## Discussion

In the present study, we demonstrated that OPTN exists mainly as oligomers, rather than as monomers, in cultured cells (Fig. 1). In addition, we showed interactions between OPTN molecules inside cells for the first time, further proving that OPTN oligomers are universal in cultured cells and showing they are localized diffusely in the cytoplasm (Fig. 3). Furthermore, this is the first study to explore the relationship between the oligomeric state of OPTN and the E50K mutation in cultured cells.

### The oligomeric state of optineurin

A previous study, using gel filtration analysis, revealed that NRP, another name for OPTN, is present in a high molecular mass complex ranging from 400 to 700 kDa [10]. Later, an experiment employing BNE showed that OPTN is able to form 420-kDa oligomers [14]. However, neither study elucidated that the oligomeric state is a basic characteristic of OPTN and is its physiological state in cultured cells. On the other hand, it is noteworthy that some proteins exist only, or primarily, in an oligomeric state, while some proteins polymerize depending on environmental conditions, such as temperature, pH, or a stimulus [46], [47]. In addition, experimental procedures may cause protein aggregation. However, in our study, we not only confirmed the presence of OPTN oligomers by BNE, but also performed *in situ* PLA, which directly visualized the interaction between FLAG-tagged and GFP-tagged OPTN in fixed cells (Fig. 3). Therefore, we have deduced that oligomerization of OPTN occurs under physiological conditions in cells, rather than being induced by an altered environment or some extracellular stimulus.

OPTN oligomers are dissociated by reducing reagent, so that a band with the size equivalent to the OPTN monomer is consistently detected by SDS-PAGE. This indicates that OPTN protein molecules interact with each other via noncovalent (possibly in addition to disulfide) bonding. H_2_O_2_ treatment of the cells resulted in partial cross-linking of the physiological OPTN oligomers via non-SS covalent bonds, which cannot be disrupted by reducing reagent, and could be detected on SDS-PAGE (Fig. 2A–C, arrows).

To determine the relationship between the noncovalent OPTN oligomers detected by BNE and the covalent OPTN oligomers observed by SDS-PAGE, we firstly compared their molecular masses. The covalent OPTN oligomers observed by SDS-PAGE were found to be trimers. Although this conclusion was drawn only based on the molecular masses estimated from SDS-PAGE, the molecular mass ratios of the covalent OPTN oligomer to the OPTN monomer were all around 3 (Table 2), in the case of both endogenous and transfected WT OPTN and the deletion mutants (Lc1st, Lc2nd, and Lc3rd). Even though the size of the WT and each deletion mutant OPTN varies, the covalent oligomer is always triple the size of its corresponding monomer, which strongly indicates that the components of the covalent OPTN oligomer are three OPTN molecules. Since SDS-PAGE can be reliably used to determine the molecular weight of proteins and we estimated molecular weights from the same gel, it is plausible that the covalent oligomers are cOPTN_3_.

In contrast to SDS-PAGE, the mass estimation of native proteins by BNE has many potential pitfalls. The charge/mass ratio of a protein was considered to define its maximum electrophoretic mobility, which is related to the estimated mass of the protein. Unlike SDS-PAGE, in which the SDS/protein ratio is usually 1.4 (g/g) and the charge/mass ratio is identical for the majority of proteins, the ratio of bound Coomassie dye/protein and the charge/mass ratio in BNE is variable, depending on individual proteins. Hence, mass estimation by BNE is not as accurate as that by SDS-PAGE and may have much larger error [48], [49]. Therefore, it is challenging to determine whether noncovalent oligomers are OPTN homo-oligomers or hetero-oligomers merely based on the size estimated from BNE (Fig. 1A).

Taken together, we propose two possible models for the oligomeric state of OPTN. In one model, the OPTN trimer is suggested to be the basic unit, two of which form a hexamer, which exists under physiological conditions in cells. Cross-linking of OPTN under oxidative stress happens between monomers within a trimer, rather than between two trimers; consequently, covalent trimers are detected by SDS-PAGE, while the bonds between two trimers are complete abrogated under reducing conditions. In another model, the OPTN trimer is proposed to be the physiologically existing form of OPTN. The BNE-detected oligomers may contain other interacting molecules. In both models, the OPTN trimer is of great importance.

Most interestingly, trimerization of NEMO, via its coiled-coil C terminal domain, has been reported [42], [50]. Our data showed that the UBD within the 4th region of OPTN is necessary for the formation of OPTN trimers. It should be noted that OPTN shares 53% amino acid similarity with NEMO and that the 4th region of OPTN has the highest homology to NEMO. The sequence similarity of these two proteins may lead to a similar oligomerization status, which increases the importance and validity of OPTN trimers as the target for analysis of OPTN oligomerization. To date, little is known about the OPTN oligomerization mechanism and its regulation and these aspects require additional study.

### New insights into E50K pathogenesis

cOPTN_3_ can be induced by H_2_O_2_ stimulation to cells. Interestingly, we found that cOPTN_3_ can also be observed in the E50K-transfected cells without additional stimuli, but is not observed in cells expressing OPTN bearing other glaucoma-associated mutations. Since there are no differences in protein expression level between E50K or the other mutants (Fig. 5, lower bands), the differences are not due to a higher level of E50K expression. Furthermore, E50K-induced cOPTN_3_ was suppressed to varying degrees by antioxidants, which together indicates that E50K induces cOPTN_3_ formation by increasing intracellular oxidative stress. Earlier studies have shown that both endogenous and exogenous WT OPTN translocate to the nucleus upon H_2_O_2_ stimulus in NIH3T3 and Neuro2a cells; the same phenomenon occurred with transfection of E50K into cells, indicating that the mere expression of E50K stresses these cells [40]. In another report, it was shown that E50K-induced oxidative stress in RGC-5 cells, playing an important role in RGC-5 cell death [29]. Our study provides further evidence that E50K causes oxidative stress to cells; moreover, for the first time, we elucidated that E50K affects the oligomeric state of OPTN by converting the type of bonds in the OPTN oligomer. These results indicated that oxidative stress plays an important role in the pathogenic mechanism of E50K. Oxidative stress is well known to contribute to the pathogenesis of glaucomatous neurodegeneration. It leads to the death of RGCs via direct cytotoxicity or by modulating protein function by oxidatively modifying the protein, for example, by “protein carbonylation” [5]. Whether E50K-induced oxidative stress has similar effects requires further investigation.

To date, the functional outcomes of cOPTN_3_ remain unclear. However, there are numerous studies demonstrating that cross-linkage of proteins by oxidative stress leads to loss and/or gain of function of target proteins. It has been shown that the cross-linked protein products induced by oxidative stress are not only resistant to degradation by the proteasome, but also inhibit the ability of the proteasome to degrade oxidized derivatives of other proteins, thereby causing a vicious cycle that leads to progressive accumulation of cytotoxic protein oxidation products [51]–[54]. From our preliminary data, we observed that the amount of cOPTN_3_ in E50K-transfected cells seemed to depend on the harvest time after transfection. More cOPTN_3_ was observed when cells were harvested two days after transfection of E50K than after one day, which suggests that cOPTN_3_ may be resistant to proteolysis and as a result accumulates in cells.

Moreover, it is well known that oxidative derivatives of specific proteins play an important role in neurodegenerative disorders. The oxidative formation of dityrosine cross-linked dimers of α-synuclein was found to be the rate-limiting step for α-synuclein fibrillogenesis in Parkinson's disease [55], [56]. The oxidation of creatine kinase may account for its decreased activity in Alzheimer's disease (AD), which may be involved in the decreased energy metabolism in the AD brain [57], [58]. Considering the mounting evidence for cytotoxicity of protein oxidation, it is reasonable to expect that cOPTN_3_, as an E50K-induced oxidation product, in turn, contributes to the pathological lesions of E50K-associated glaucoma. The relationship between these is likely to become an important focus for research in the near future.
